# Supplementary material for: TRIM28 is a target for paramyxovirus V proteins
Source: PLoS Pathog. 2025 Sep 8;21(9):e1013487. doi: 10.1371/journal.ppat.1013487 (PMC12431652; doi:10.1371/journal.ppat.1013487)
Supplement: Attachment — Submitted filename: 12_T28V_ResponsetoReviewers.docx [file ppat.1013487.s005.docx]

**Author response letter for PPATHOGENS-D-25-01143**

*> We thank the two reviewers and the editor for their thoughtful and constructive comments on our manuscript. Please find below the points raised, with our responses in blue italics (line numbers refer to the tracked-changes version of the manuscript) .*

**Editor:**

Most of the comments can likely be addressed with text responses and minor changes, though I would suggest experimentally addressing the following critiques:

1. Reviewer 1 requests evidence that V/TRIM28 interaction is not a post-lysis artifact given the disparate localizations of the two proteins.
2. Co-infection experiments requested by Reviewer 1. IAV infection of V-transfected cells may be the most straightforward experiment.

*> We thank the editor for clarifying the major issues. We have now performed additional experiments that address these comments and have included the new results in Figures 1 and 5. The new data support the original hypothesis and strengthen the manuscript as a whole (see specific responses to reviewers below).*

**Reviewer #1:**

**Summary**.

The authors show a novel interaction between TRIM28 and the paramyxovirus V protein. Using coIPs, the authors show interaction between TRIM28 and the V proteins of several paramyxoviruses. Furthermore, the authors map the interaction between PIV2 and PIV5 V proteins and TRIM28 to the C-terminus of V and the domains of TRIM28 interacting with V. Point mutations are then generated to test the importance of specific residues necessary for TRIM28-V interactions. A few issues need to be addressed.

*> We thank the reviewer for highlighting the novelty of our study.*

**Major Issues**.

Figure 1. (applies for all CoIPs).

- TRIM28 is thought to be localized to the nucleus and V protein is localized to the cytoplasm. It would be important to isolate nuclear and cytoplasmic fractions separately and not total cell lysates to show that the interaction observed between TRIM28 and V is not an artifact of the lysis procedure.

*> This is an important point that we also struggled with initially, as we also thought that TRIM28 is localised to the nucleus, while V proteins are localised to the cytoplasm. However, V localisation to the cytoplasm is only generally assumed from its known functions in interacting with cytoplasmic factors such as mda-5 and STAT proteins. A more in-depth review of the existing literature reveals that there is already a great body of evidence indicating that V proteins can localise to the nucleus, where they might engage nuclear TRIM28. For example, indirect immunofluorescence and cellular fractionation studies have shown that PIV2-V (when expressed alone or in the context of infection) can be present in both the cytoplasm and nuclei of cells (PMIDs:* ***8627237; 12388709). Similarly, PIV5-V and MeV-V have been described in both the nucleus and cytoplasm, with indirect immunofluorescence studies indicating that MeV-V is actually predominantly nuclear (****PMIDs:* ***22532687; 12805463****;* ***11831692****). It is, however, worth noting that some paramyxovirus V proteins are mainly cytoplasmic in intracellular distribution during the steady-state, such as MuV-V, MapV-V and NiV-V (PMIDs:* ***22532687; 17325370; 12388709). Nevertheless, experiments with the nuclear export inhibitor, leptomycin B, have provided clear evidence that NiV-V protein is a shuttling protein that transiently enters the nucleus (PMID: 12388709****). This history of literature therefore provides solid and independent evidence for the nuclear localisation of many paramyxovirus V proteins, and we have updated the text and references throughout the manuscript to make this clearer to the reader and highlight this ‘forgotten’ observation (lines 165-168, 422-423). Nuclear localisation of V proteins is therefore entirely compatible with its engagement of TRIM28.*

*> In addition, to improve the manuscript and to add our own experimental data, we have now also performed new indirect immunofluorescence microscopy analyses on V transfected cells. This analysis of representative V proteins with a spectrum of TRIM28 engagement activities revealed that a substantial fraction of each V does indeed reach the nucleus where TRIM28 is primarily located. This is consistent with the studies of others detailed above. We also immunostained for TRIM28 and can show that TRIM28 levels and localisation do not change with V transfection. These new data are presented in Figure 1E (lines 168-171) and support the concept that V proteins do localise to the nucleus where they can engage TRIM28. Interestingly, we note that some of the V proteins we identified to poorly interact with TRIM28 are also poorly identifiable in the nucleus under steady-state conditions (e.g. NiV-V), which is consisten with the work of others demonstrating a transient nuclear-shuttling function* ***(PMID: 12388709****). We have commented upon the possible meaning of this in the text (lines 173-175), along with the observation that the region of PIV2-V important for determining V-TRIM28 interaction has previously been shown by others to determine PIV2-V nuclear localisation and retention (PMID:* ***8627237)*** *(lines 206-207, 232-234, 484-486).*

Figure 2.

- Panel 1A shows MeV V migrating close to the 37kDa marker. However, in Figure 1, MeV V migrates close to the 25kDa marker. Please explain and clarify if this is a mistake.

*> We thank the reviewer for noticing this error. Indeed, the markers in the original Figure 1A were not labelled correctly (a general template was used when putting the figure together, but the markers were not adjusted to be correct). We apologise for this oversight and have provided an updated figure 1A with the markers now correctly labelled.*

- Panel 2B - mCherry migrates at a different position compared to Figure 1A. The migration pattern between mCherry, PIV2, and PIV5 is similar, but the marker is not. Please clarify if figure 1 is correct.

*> See answer above about the original error in Figure 1A labelling.*

- Panel 2E, G, I - Plotting relative co-IP on different axis types (log vs linear) prevents the reader from easily comparing results between panels. Please use a uniform axis type.

*> Graphs in these 3 panels have now been updated to be consistent in their y-axis type.*

Figure 3.

- Since the similarity between TRIM28 across species is highly conserved, it would be useful to include locations of non-conserved residues between species as part of the schematic. According to the sequence identify, there should only be 5-15 changes between humans and mice. would be easiest to show as a sequence alignment in supplement.

*> We thank the reviewer for this suggestion and have now included the sequence alignments as new Supplementary Figure 1.*

- Panel B,C, and E - MeV V is above 37kDa. However, in Figure 1a, MeV V is close to 25kDa. Explain.

*> See answer above about the original error in Figure 1A labelling.*

Figure 4.

- Panel E and F - How can you make error bars representing Standard Deviation using an N of 2? Please change to range.

*> We have updated the graphs in these 2 panels to now indicate range.*

Co-infection studies.

- In addition to coinfections, it would be very important to show the effects of PIV2/PIV5 coinfection with influenza are due to the V protein or a different viral protein. Transfection of cells with V protein expression plasmids, followed by infection with influenza would greatly strengthen the arguments proposed by the author if the results are the same. Particularly when it comes to SUMO2/3 activity and ERE activation/RNA levels. One more thing, the authors have a delta V PIV5 virus. It would be highly relevant to include this virus with the coinfection studies to show the effects of delta V PIV5 on both SUMO2/3 activity and ERE activation.

*> The reviewer makes several astute comments, and highlights areas that we initially had technical difficulties with addressing prior to the first submssion. Regarding potential use of PIV5VdelC, it is technically and conceptually problemmatic for two reasons:*

*(1) PIV5VdelC, as compared with wild-type PIV5, is a powerful inducer of IFN during infection, meaning that effective subsequent infection with IAV is difficult. Without comparable IAV infection levels between PIV5VdelC and wild-type PIV5 conditions, the effects on ERE activation would be impossible to fairly assess.*

*(2) In Figures 5B/5F, we found that wild-type PIV5 was unable to restrain IAV-mediated loss of SUMO-modified TRIM28, which may be due to the comparatively low efficiency of PIV5-V engagement with TRIM28 (Figure 1B). Thus, if no ‘antagonistic’ effects could be noted with wild-type PIV5, we reasoned that we could not observe any reversal with PIV5VdelC. Unfortunately, we do not have access to a PIV2VdelC virus, that would have been the ideal control companion to wild-type PIV2.*

*> Nevertheless, to address the reviewer’s specific comment to show that the effects noted are likely due to PIV2-V, and not another PIV2 protein, we have now performed the suggested experiment involving transfection of PIV2-V (or a control) into HEK293T cells followed by assessment of subsequent TRIM28-dependent ERE expression induced by IAV infection. The results now make up new Figure 5I-J (with discussion on lines 391-393, 428-430), and complement the original co-infection studies nicely by indeed showing the PIV2-V alone is similar to PIV2 infection in limiting expression of IAV-induced HERVK14C. We thank the reviewer for suggesting this experiment, as we believe it has greatly improved the manuscript and clarity of findings.*

**Minor Issues**.

Figure 5.

Fluorescence microscopy showing the localization of PIV2/PIV5 V and TRIM28 would greatly strengthen the manuscript.

*> As part of our response to the reviewer’s earlier comment relating to localisation of V and TRIM28 interaction, we have now added new indirect immunofluorescence microscopy images as Figure 1E, which indeed strengthens the manuscript.*

**Reviewer #2:**

**Summary**.

The manuscript by Lieber et al. characterizes a novel TRIM28 binding function by the V protein of different paramyxoviruses. TRIM28, when SUMOylated can repress transcription of endogenous retroelements, which can have an impact on antiviral immunity, as in the case of influenza A virus, as described by the authors. Here, they described the TRIM28-V protein interaction and identify key regions of the V protein and TRIM28 where the interaction occurs. Interestingly there are differences in TRIM28 affinity depending on which paramyxovirus V protein is tested, the implications of which are not completely understood and can be further studied. Also of note was that this interaction did not lead to loss of ERE repression that is seen during influenza infection. Pre-infection with PIV-2, whose V protein has a strong interaction with TRIM28, prevented the loss of TRIM28 SUMOylation seen during subsequent influenza infection, significantly impacting ERE transcription.
The authors present their story in a compelling fashion and in a logical step-wise manner that is easy to follow and coherent. They convincingly show that V-TRIM28 interaction occurs and through specific C terminal domains in the V protein interacting with CC and PHB domains of TRIM28. The authors bring up several current unknowns and possible areas for future study in the discussion. I have only minor comments and questions for the authors*.*

*> We thank the reviewer for highlighting the compelling and convincing nature of our study and its logical presentation. We address their minor comments and questions below.*

**Major Issues**.

No response.

**Minor Issues**.

For the initial experiments, the authors perform coIP at 24 hours post-transfection and post-infection. During infection, what are the kinetics of V protein expression? Would it be of interest to examine how early V is expressed and begins to interact with TRIM28? Conversely, particularly during infection, which may last days, how stable is the V-TRIM28 interaction and for how long can EREs remain repressed? Presumably new V is produced during infection of new cells and continues this effect. The authors use a high MOI to produce infection in a high % of cells. But if a lower MOI is used and a small fraction of cells are infected initially, does the stress response in infection cells lead to derepression of EREs and possible innate immune stimulation in neighboring, uninfected cells that may negate the impact of this effect in infected cells?

*> These are certainly important points that we take note of, and we are grateful to the reviewer for raising them. These are questions that we envisage could be addressed in future studies to uncover the nuances of the observed responses, and may be (in our opinion) beyond the scope of the present study.*

In fig 1 the anticipated sizes of the V proteins of various paramyxoviruses varies considerably with HeV, NiV, and SeV are approximately 2x as large as others. Is this expected? This is my naivety with respect to different viruses in the family.

*> Indeed, untagged paramyxovirus V proteins range in predicted size from about 24kDa (e.g. PIV5-V/PIV2-V) to about 50kDa (e.g. HeV-V/NiV-V). The sizes as shown in Figure 1 are as expected.*

For figure 4, are C and D simply replicates of the same experiments? There are no differences between the panels other than the blot bands, but it is not explicitly stated anywhere that this is the case, so it was a bit confusing. The same for Fig 3 C and D. these are different domains being tested so that can be gleaned from looking at the labels, but it is not obvious and not obvious from the figure caption either.

*> We have now clarified these discrepancies in the two legend texts. Figure panels 4C and 4D are different infections with PIV2 and PIV5, respectively, which, while noted on the figure itself, was not stated clearly in the legend in the first submission. For figure panels 3B and 3C, these do indeed involve different domains, and we have now referred the reader to the terminology in panel 3A to clarify this in the legend text.*

For figure 3, the experiments were done in TRIM28 KO cells but for the experiments with TRIM24, 33, 66 were these also knocked out? Since you’re only looking at the specific domains, it is less of a concern that what you’re seeing in the blot is FL protein as a false positive, however this may impact V-TRIM interactions.

*> We have now clarified this in the text on lines 281-283 to address the caveat that we do not have an appreciation of the contribution of the full-length proteins in this experimental system.*

In the methods for influenza infection, the authors do not indicate if they used exogenous trypsin to aid in infection. They use a high MOI and look at the effects shortly after infection, so it is probably not an issue because they don’t need efficient replication of the virus. However a statement about this could be added.

*> Details on TPCK-trypsin addition to the IAV infection conditions are now detailed in the methods on lines 528-529.*

The authors interestingly note that V proteins are usually cytoplasmic, and this interaction with nuclear proteins is novel. Is anything known about nuclear localization of V proteins? The authors mention previous studies showing it in the nucleus but with no known function. Is the C terminal domain critical also for nuclear localization? The authors could look at how it locates and important domains for this. They touch on this and speculate but did not explore it experimentally.

*> This important point was similarly raised by reviewer 1, and we have added new experimental results addressing this as Figure 1E, together with associated text in the results/discussion. Please see response to reviewer 1, major point 1 and minor point 1.*

The authors do not actually look at any innate immune signaling that may or may not be triggered by transcription of EREs in their transfection or infection systems. This would be an important downstream effect that could shed light on outcomes of infection, at least in vitro.

*> This is certainly an interesting point, however it is extremely difficult to directly dissect the impact of ERE upregulation on innate immune signaling, particuarly in the context of an ongoing viral infection that may be stimulating innate immune pathways by alternative mechanisms (e.g. via viral RNA) or indeed inhibiting innate immune signaling by targeting things such as RIG-I-like receptors or JAK-STAT pathways. We believe that the only way to ultimately link EREs to innate immune activation will be to understand the specific viral trigger of EREs in more depth, and then activate EREs in the absence of infection, or to understand the specific pathway leading to ERE activation and be able to inhibit this pathway chemically or genetically during infection. Currently, such studies are beyond the scope of the present study.*

To me the most interesting finding is regarding NiV V and how it’s TRIM28 precipitation was less efficient, though it would probably be thought of as the most virulent of these viruses in humans. This is raised by the authors in the discussion however it seems to me that if this is the case, then during infection of hosts (particularly humans or other susceptible animals) that even if V is suppressing ERE transcription and this does have an impact on innate immunity, the effect likely is not impacting infection outcomes in the host and plays only a very minor role. It also could be that other innate immune signaling via other means dwarfs that being induced by EREs. Something interested to pursue further.

*> We agree with the reviewer that this is an interesting area to consider, as many of these paramyxoviruses could be considered to be adapted to different host species and engage differentially with species-specific factors. As virulence is multifactorial, it is difficult to assign any specific function of V as contributing to susceptibility. Of course, abilities to suppress ERE responses, and potentially consequent immune responses, could have various roles. For example, while we primarily hypothesise that ERE upregulation is a host defence mechanism that limits viral spread, and therefore viral suppression of this response might benefit viral replication and subsequently enhance disease, it may also be that failure to suppress the ERE response by some viruses leads to hyperinflammatory state that also enhances viral/immunopathological disease without benefiting viral replication. One might speculate that this could be a consequence in the case of NiV-V failing to engage TRIM28, leading this virus infection to be particularly virulent due to enhanced immunopathology. While these are interesting and important points, we do not wish to be too speculative in our discussion, and leave these aspects of the outlook for future work.*
